# Evaluation of the Frails' Fall Efficacy by Comparing Treatments (EFFECT) on reducing fall and fear of fall in moderately frail older adults: study protocol for a randomised control trial

**DOI:** 10.1186/1745-6215-12-155

**Published:** 2011-06-18

**Authors:** Boon Chong Kwok, Kaysar Mamun, Manju Chandran, Chek Hooi Wong

**Affiliations:** 1 Department of Physiotherapy, Singapore General Hospital, Outram Road, Singapore; 2 Department of Geriatric Medicine, Singapore General Hospital, Outram Road, Singapore; 3 Osteoporosis and Bone Metabolism Unit, Department of Endocrinology, Singapore General Hospital, Outram Road, Singapore; 4 Department of Geriatric Medicine, Khoo Teck Puat Hospital, Yishun Central, Singapore

## Abstract

**Background:**

Falls are common in frail older adults and often result in injuries and hospitalisation. The Nintendo^® ^Wii™ is an easily available exercise modality in the community which has been shown to improve lower limb strength and balance. However, not much is known on the effectiveness of the Nintendo^® ^Wii™ to improve fall efficacy and reduce falls in a moderately frail older adult. Fall efficacy is the measure of fear of falling in performing various daily activities. Fear contributes to avoidance of activities and functional decline.

**Methods:**

This randomised active-control trial is a comparison between the Nintendo WiiActive programme against standard gym-based rehabilitation of the older population. Eighty subjects aged above 60, fallers and non-fallers, will be recruited from the hospital outpatient clinic. The primary outcome measure is the Modified Falls Efficacy Scale and the secondary outcome measures are self-reported falls, quadriceps strength, walking agility, dynamic balance and quality of life assessments.

**Discussions:**

The study is the first randomised control trial using the Nintendo Wii as a rehabilitation modality investigating a change in fall efficacy and self-reported falls. Longitudinally, the study will investigate if the interventions can successfully reduce falls and analyse the cost-effectiveness of the programme.

**Trial registration:**

Australia and New Zealand Clinical Trials Register (ANZCTR): ACTRN12610000576022

## Background

In Singapore, there is an increasing growth of older adults (300,000) and the prevalence of falls is high with a reported incidence of 129000 per year by the Singapore Ministry of Health in 2007. The common consequences of these falls are fractures and soft tissues injuries that may lead to significant disability [1]. Fractures managed in an acute care setting contribute to rising healthcare cost [2-5]. In addition minor trauma and soft tissue injuries lead to activity avoidance and embeds the fear of further falling psychologically [6].

A previous experience of fall may subsequently cause fear in the older person who will then self-restrict mobility or will depend on others for activities of daily living (ADLs) [7]. Fear of falling significantly contributes to functional decline [7] and the health and well-being of the older person [8-10]. In order to quantify the fear of fall, fall efficacy scales have been developed to assess the perceived confidence of the older population [11-13].

Contribution to frailty is a history of sedentary lifestyle in the older population which results in strength and balance impairment [7,14-16]. There are numerous interventions in rehabilitation to modify fall risk and reverse these impairments. There are many research that addressed rehabilitation in the frail population [16-24]; however those that improve fall efficacy significantly are scarce [25]. The Nintendo Wii system has been postulated to be effective in improving functional performance by enhancing flexibility, balance, strength and coordination training [26]. The additional advantage of the Nintendo Wii system is the visual feedback that the participant receives during the training session. This visual feedback aids the participants to improve balance [27], while the game simulation creates a perception that they can perform high level activities.

## Objectives

### Primary research hypothesis

The Nintendo Wii group intervention is more effective than the gym-based group intervention in improving fall efficacy and self-reported falls in the older adult aged 60 and above.

### Secondary research hypothesis

The Nintendo Wii group intervention is more effective than the gym-based group intervention to improve the secondary outcome measures: knee extension strength, timed up and go (TUG) test, 6-minute walk test (6MWT), narrow corridor walk (NCW) test, gait speed, Short Form -- 36 version 2 (SF-36v2) questionnaire and European Quality of Life -- 5 dimensions (EQ-5D) questionnaire.

## Methods

### Design

**Figure 1:**
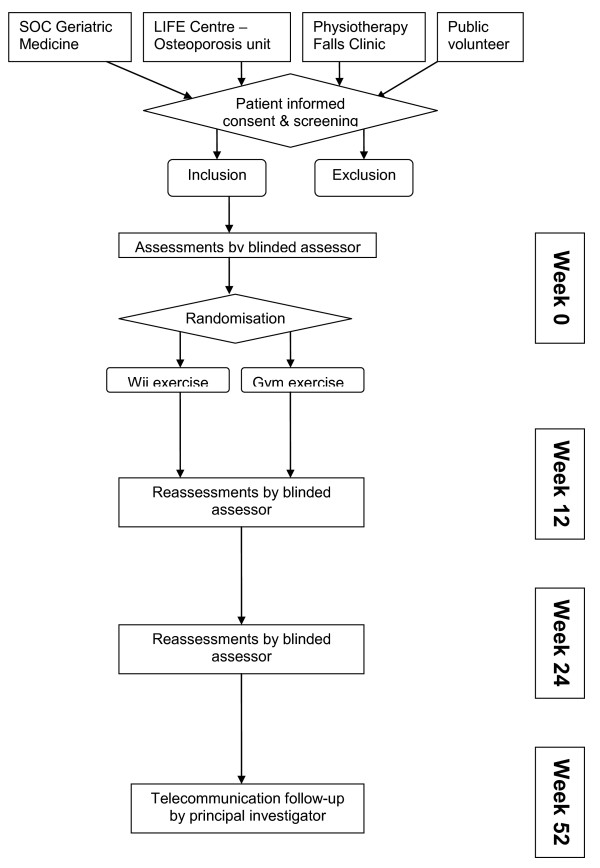
**Participant flow**. SOC: Specialist Outpatient Clinic LIFE: Lifestyle Improvement Fitness Enhancement

This is a parallel prospective single-blind randomised active-control trial. The ratio of subjects in each group is 1:1; refer to Figure 1 for the flow of participants in our study (week 0 to week 13). Each participant who completes the week 13 follow-up will be given a pedometer to track daily physical activity from week 13 to 24 and a final assessment is conducted on week 24. During the first 13 weeks, participants report weekly on incidence of falls to the interventionist. From week 14 to 52, participants record incidence of fall on a falls history sheet kept at home and to report the falls to the Principal Investigator or the outcome assessor immediately. The outcome assessor will contact the participants monthly from week 14 to 52 to update on any fall incidence.

### Participants

The participants are patients from the Singapore General Hospital (SGH) Specialist Outpatient Centre Geriatric Medicine department, SGH Lifestyle Improvement Fitness Enhancement Centre Osteoporosis and Bone Metabolism unit, SGH Physiotherapy Falls Clinic and public volunteers. The volunteers are approached to participate in the study within the hospital premises. The screening of eligible participants is done by a medical doctor or a physiotherapist during consultation sessions. The abbreviated mental test is used to screen for cognitive impairment [28], while the Short Physical Performance Battery (SPPB) is used to stratify frailty [29]. The scores from the AMT and SPPB include or exclude participants from the study.

The inclusion criteria of this study:

• Age 60 years old and above

• Community dwelling

• Independent in ambulation (with or without walking stick assist)

• Have fear of falls in daily activities

• Able to commit 12 weeks of supervised intervention

• Score of SPPB from 5 to 9 (moderately frail)

• Speaks or understands English, Mandarin or local dialects

The exclusion criteria of this study:

• Permanent bedbound status

• Disabled in ambulation

• Significant cognitive disorder (AMT score below 7 for 60-74 years old, below 6 for 75 years old and above)

• Untreated medical conditions

• End-stage illness or disease and/or life expectancy less than a year

• Unstable cerebral haemorrhage in the past 3 months

• Fractures in healing phase

• Surgery in the past 3 months, unless with surgeon's approval

• Pulmonary embolism or deep vein thrombosis in the past 3 months

Participants who are eligible and agree to register for our study will be assessed in the SGH Physiotherapy department for baseline measures by an independent outcome assessor.

### Ethics and withdrawals

This study has been approved by the SingHealth Centralised Institutional Review Board D (Reference: 2010/177/D) in accordance with the International Conference on Harmonisation (ICH)/Singapore Guidelines for Good Clinical Practices (GCP). Written informed consent is obtained from the participant using the forms from the ICH/Singapore Guidelines for GCP. The principal investigator of our study is Boon Chong Kwok, a physiotherapist with Singapore General Hospital.

Participants have the right to withdraw from the study at any time during the trial, but are encouraged to complete the trial after randomisation to minimise drop-out rate. In the event of adverse change of medical condition in the participant deemed unsafe to continue with the study by the principal investigator or co-investigators, the trial for the participant will be terminated.

### Interventions

The interventions are carried out at a satellite clinic to prevent unblinding of the outcome assessor. The two interventions are on separate days to minimise the chance of interaction between the participants from the different groups. The interventions were developed through an evidence-based approach. A physiotherapist with 3 years of clinical experience and American College of Sports Medicine clinical exercise specialist certification conducts the interventions for both groups with adherence to the intervention protocol.

The home exercise programme is equivalent in both groups. Exercise bands for resistance training at different difficulty levels are issued to each participant according to the baseline assessments for the first 6 weeks and the next level of band is issued on the seventh attendance.

One of the unique features of the study is that it has a component of participant specific management in both intervention arms. This enables achievement of the participant's goals in rehabilitation. Every participant may present with different impairments that will need to be addressed with specific strengthening exercises, balance and coordination training, agility training or acute pain management.

#### Novel intervention arm

The Nintendo Wii programme is incorporated with exercises that are taught as home exercises. In this study, the WiiActive game is used that includes exercise band resistance training, balance and coordination training, calisthenics, and cardiovascular training. The duration of a session is one hour including the rest interval, with the following breakdown: 10 minutes of stretching exercises, 20 minutes of WiiActive game play, 15 minutes of home exercises education, and 15 minutes of participant specific management.

#### Traditional intervention arm

Supervised exercises in a gym setting include resistance band training, balance and coordination training, calisthenics, and cardiovascular training. The duration of a session is one hour including the rest interval, with the following breakdown: 10 minutes of stretching exercises, 20 minutes of cardiovascular training, 15 minutes of home exercises education, and 15 minutes of participant specific management.

Participants are to attend once per week of supervised exercises and carry out home exercises at least twice per week. Exercise log sheets and home exercise handouts are issued to each participant on the first intervention session and the log sheets are collected on the last intervention session. Compliance to the programme is monitored by the attendance rate [30].

Home safety and modification advice is provided in a handout for each participant [31]. The details of the handout are shown in Table 1.

**Table 1:** Home safety and modification

Locations	Modifications
Living room	Ensure that wires and cords are out of the way.
	
	Arrange furniture so that you can move around easily and safely.
	
	Remove carpets and rugs that are along your walking path.

Kitchen	Try to make sure that items are easily accessible to avoid reaching for them.
	
	Wires and cords should not be in your path.
	
	Clean all spills immediately.

Self-care	If you wear shoes at home, use rubber-soled shoes.
	
	Ensure you use a walking aid as necessary.
	
	Make sure you go for regular medical and eye check-ups.
	
	Take your medications as prescribed.
	
	Be aware of the side-effects of the medications you are taking.

Bathroom	Install grab rails on walks.
	
	Place non-slip mats in the shower area.
	
	Use a shower chair and a portable shower head when showering.
	

Bedroom	Place light switches within reach of your bed.
	
	Have night lights between the bathroom and the bed.
	
	When getting out of bed, sit and wait a while before standing up to avoid getting dizzy.
	

Stairways	Keep stairway free of clutter.
	
	Install handrails on one or both sides of the stairs if possible.

PPA: Physiological Profile Assessment, SPPB: Short Physical Performance Battery, MFES: Modified Fall Efficacy Scale, Short FES-I: Short Fall Efficacy Scale -- International, 6MWT: 6-minute walk test, TUG: Timed-up-and-go, NCW: Narrow corridor walk, SF-36v2: Short form -- 36 version 2, EQ-5D: European Quality of Life -- 5 dimensions, GROC: Global rating of change

### Baseline data

Demographics are obtained via the assessor on the first assessment session. Baseline demographics include age, gender, ethnic group, living status (staying alone or with family), history of falls in the past 12 months, number of medications, height, weight, resting heart rate, resting blood pressure, AMT, SPPB score and Physiological Profile Assessment (PPA) fall risk score.

### Outcomes

The outcome measures are assessed one week before the first intervention session (week 0), one week after the last intervention session (week 13) and a final reassessment on week 24 by an independent blinded outcome assessor (Table 2). The primary outcome of this study is an improvement in fall efficacy with the Modified Falls Efficacy Scale (MFES). New recent falls after the initiation of intervention will investigate the success of the programme in falls prevention. This is carried out by self report of falls by the participants via telecommunication and on a falls recording sheet. The secondary outcomes of this study are improvement in knee extensor strength, endurance, agility, functional status and quality of life. The global rating of change (GROC) scale is used to assess the participant's perceived change from baseline on each follow-up [32,33].

**Table 2:** Data collection time-points

Data	Baseline	Week 13	Week 24	Week 52
Demographics	**X**			
Fall history	**X**	**X**	**X**	**X**
Fall risk (PPA)	**X**	**X**		
Frailty (SPPB)	**X**	**X**		
Fall efficacy (MFES, Short FES-I)	**X**	**X**	**X**	
Strength (knee extension)	**X**	**X**	**X**	
Endurance (6MWT)	**X**	**X**	**X**	
Agility (TUG)	**X**	**X**	**X**	
Dynamic balance (NCW)	**X**	**X**		
Gait speed (4 m walk)	**X**	**X**	**X**	
Quality of life (SF-36v2*, EQ-5D)	**X**	**X**	**X**	
Participant's perceived change (GROC)		**X**	**X**	
Home exercises (Exercise diary)		**X**		
Physical activity (Pedometer)			**X**	

*Week 24 SF-36v2 components assessed are physical function and bodily pain

Fear of fall (self efficacy): MFES is a validated 14-item questionnaire of fall efficacy of the older person in various ADLs and community activities [11]. It is administered by an interviewer approach in our study to prevent misinterpretation by the participant and to maintain consistent and accurate responses from the participant. The Short Fall Efficacy Scale -- International questionnaire takes a shorter time to administer with 7-item on indoor and outdoor activities [13]. The questionnaires have no risk of recall bias as they require the participants to rate their perceived confidence based on the day of assessment.

Strength (impairment): Knee extension strength measurement with the PPA is a maximum isometric contraction on a spring gauge [34]. The test is conducted 3 times with a rest interval of 30 seconds. Another strength measurement is the weight lifted for 10 repetitive maximum knee extension of 0 to 90°.

Endurance (functional limitation): 6MWT is one of the most commonly conducted field tests and is utilised in numerous research trials for the evaluation of aerobic capacity [35]. The test is carried out on a clear and quiet 10-metre corridor marked with a cone at each end. Instructions are given prior to the test and the time left to the end of the test is announced by the assessor at interval of 1 minute.

Functional status (functional limitation): TUG is an established test of agility in the older population and is used in this study for agility changes [36-38]. Subject is to rise from the chair and walk 3 metres, marked by a cone, and return to the seat. Higher functional task is assessed in our study using the NCW test -- a test of dynamic balance that comprises of a 6-metre walk within the width of 0.2-metre throughout the entire course [39]. Gait speed is calculated from 4-metre walk test of SPPB [15,29], which is potentially a sixth vital sign [40].

Quality of life (disability): Quality of Life will be measured with the SF-36v2 [41] and EQ-5D questionnaires [42].

### Follow-ups

On the 13^th ^week, each participant undergoes an outcome assessment. After the assessment, the participant will be issued a pedometer (HJ-109 Omron Healthcare, Japan) for daily quantifying of physical activity via number of steps taken for the day [43]. Participants in both groups will continue with the home exercises after the last supervised exercise session. The pedometer is used to gauge and encourage physical activity of the participants. A follow-up monitoring of change on the 24^th ^week will assess changes in self efficacy, impairments, functional changes and quality of life. Monthly from week 14 to 52, the participants will be contacted for any events of fall. The pedometer reading will be correlated with the change in outcome measures from week 13 to 24.

### Sample size

Our primary endpoint is a change in MFES scores immediately post-intervention. There was no literature of MFES investigated in the moderately frail population. We estimated the minimum clinically important difference (MCID) to be detected between the novel intervention programme and the traditional supervised exercise programme and derived a change in 1.5 MFES units. Previous randomised controlled trial showed no change in MFES scores post intervention because healthy volunteers were recruited in their study [25]. The use of healthy volunteers resulted in a ceiling effect in that trial. In the absence of trials assessing MFES in the moderately frail older adults, we assumed a common between-participant standard deviation of 2 units for MFES [25]. This translates to a standardized effect size of interest (Cohen's ***d***) of 0.75. Accordingly, the required sample is 30 participants per group for a two-tailed comparison of the groups when ***d ***is 0.75, power is 0.8 and type I error is 0.05. Considering a 15% drop-out rate, 80 participants will be recruited.

### Randomisation

Block randomisation will be generated by an independent investigator who is not involved in the outcome assessments and interventions and concealed in sealed envelopes marked 1 to 80. Participants will be randomised after completion of the initial assessment independently by an investigator who is not involved in the outcome assessments.

### Blinding

Due to the particular nature of our study, only the outcome assessor is blinded. Participants are not blinded in the study.

### Inter-rater reliability

Reliability of the MFES [11], Short FES-I [13], PPA knee extension [34], TUG [44], 6MWT [45], SF-36v2 [46] and EQ-5D [47] have been established. However, we find no literature on the reliability of NCW test and decided to carry out a reliability assessment.

Test-retest reliability was then established between the outcome assessors, Intraclass Correlation Coefficient 0.972, from the analysis of 10 repeated timed walk of the NCW test.

### Statistical methods

Missing data will be analysed by intention-to-treat approach. Baseline measures for age, body mass index, MFES, TUG test, 6MWT, PPA knee extension strength, NCW test, SF-36v2 and EQ-5D will be analysed for normality. The primary outcome measure is the change in MFES score. Outcome measures will be quantitatively analysed with the mixed model for between group comparisons for statistically significant difference. Statistical significance is set at *p*(2-tailed) < 0.05. Clinical significance will also be analysed based on MCID or minimal detectable change for each of the outcome measures. The mean MCID will be obtained from participants who have a rating of 3 on the GROC scale.

### Health economic evaluation

The trend of rising healthcare cost is a concern to the population. Evaluation of the cost-effectiveness of the programme will determine the feasibility of implementing the programme as a fall prevention programme. The cost-effectiveness of the intervention programmes will be analysed at the end of one year follow-up. We will analyse the cost of programme, EQ-5D, fall history and incurred opportunity cost. The EQ-5D will provide the study with Quality-Adjusted Life Years. Fall history and accompanying opportunity cost will be obtained from telecommunication interview with the participant. Opportunity cost of a fall is equivalent to the total cost of treatment and management for the fall. The limit for overall cost per fall prevented is set at S$20,000 to evaluate the programme cost effectiveness.

### Time-line

The study time-line is monitored by the study principal investigator. Table 3 reflects the milestones of the study.

**Table 3:** EFFECT milestones

Time-line	Events
Jan 2009	Gym-based group exercises for the older population developed
Jul 2009	Randomised controlled trial planned
Aug 2009	SHF Research grant submitted
Feb 2010	Research grant awarded
Jun 2010	Study commenced
Jul 2010	Recruitment commenced
Mar 2011	Recruitment completed
Jun 2011	Last 12-week follow-up
Sep 2011	Last 24-week follow-up
Oct 2011	Data analysis completed
Nov 2011	Publication submitted

SHF: SingHealth Foundation

## Discussion

The EFFECT study is the first randomised active-control trial evaluating the reduction of fear of falling and self-reported falls between the Nintendo Wii and the standard intervention. The intervention in this study undertakes a multimodal approach in rehabilitation that is in accordance with evidence-based practice [16,18-20,22,24,48].

The WiiActive game is beneficial for the older person with strength, cardiovascular, balance and coordination exercises [17]. The band exercises may be more readily accepted as compared to stack weights and free weights. Majority of the older people are frail and cannot jog in the WiiActive game. Modified instructions to march on the spot will allow them to complete difficult exercises and improve confidence. Balance training with the WiiFit board is achieved through activities that require the participant to avoid obstacles in activities such as roller-skating. Progression to a combination of balance and coordination training that involves upper limb and lower limb movements is practised with virtual tennis simulation.

In the clinical setting, the potential use of gaming with visual feedback can facilitate the reduction of physical time that the therapist has to spend with the patient. Short-term visual feedback rehabilitation has also been found effective in improving functional status [27]. Furthermore, the exercises when done in a group can enhance compliance and improve motivation [49].

The minor disadvantage of the WiiActive game is the requirement the controller be held by the participant for the system to register movement patterns. This can be overcome if game developers utilise the WiiMotion-Cam technology that allows a controller-free interface.

The unique feature of the group exercise programme is the component of specific modalities for participant based on the initial assessment. This is to ensure that the major issues and goals raised by the subjects are treated and achieved within the 12 weeks of intervention.

Our study aims to achieve fall prevention and improving confidence of older adults in performing ADLs. The study investigates if the use of virtual feedback gaming will achieve these outcomes superior to a standard rehabilitation model.

## Abbreviations

ADLs: Activities of daily living; TUG: Timed up and go; 6MWT: 6-minute walk test; NCW: Narrow corridor walk; SF-36v2: Short Form -- 36 version 2; EQ-5D: European Quality of Life -- 5 dimensions; SGH: Singapore General Hospital; SPPB: Short Physical Performance Battery; ICH: International Conference on Harmonisation; GCP: Singapore Guidelines for Good Clinical Practices; PPA: Physiological Profile Assessment; MFES: Modified Falls Efficacy Scale; GROC: Global rating of change; MCID: Minimum clinically important difference;

## Competing interests

The authors declare that they have no competing interests.

## Authors' contributions

All the authors are involved in the trial design discussion and study protocol development.
